# Acceptability of patient education for hypertension self-management among healthcare providers and beneficiaries in South Africa, 2024: A qualitative study

**DOI:** 10.4102/phcfm.v17i1.4801

**Published:** 2025-06-23

**Authors:** Xoliswa Simelane, Juliana Kagura, Athini Nyatela, Samanta T. Lalla-Edward

**Affiliations:** 1 Division of Epidemiology and Biostatistics, Faculty of Health Sciences, University of the Witwatersrand, Johannesburg, South Africa; 2 Ezintsha, Faculty of Health Sciences, University of the Witwatersrand, Johannesburg, South Africa

**Keywords:** acceptability, patient education, TFA, self-management, hypertension, PLWH, healthcare providers, qualitative

## Abstract

**Background:**

The prevalence of hypertension (HTN) is increasing among people living with human immunodeficiency virus (HIV). Self-management is vital for improving health outcomes and preventing disease progression. While education for HTN self-management has been implemented in South Africa, little is known about its acceptability.

**Aim:**

The study aims to explore the acceptability of patient education for self-management of HTN among people living with HIV (PLWH) and HTN, and healthcare providers in Integrating HIV and hEART health in South Africa (iHEART-SA) intervention clinics.

**Setting:**

The study was conducted in six primary health care facilities in Johannesburg.

**Methods:**

A qualitative study design using in-depth interviews (IDIs) was conducted with 18 healthcare providers and 13 PLWH and HTN. Data were gathered using a semi-structured interview guide. Interviews were conducted in English and audio recorded for transcription and analysis. MAXQDA was used for analysis.

**Results:**

The use of simple language, empowerment through knowledge and perceived health improvements were identified as facilitators of acceptability. Emotional discomfort attributable to booklet colours, diagnosis denial and staff shortages, were barriers. Ongoing training was the preferred strategy to enhance acceptability.

**Conclusion:**

Acceptability of patient education among participants was high and can be enhanced via continuous trainings. Future research should further explore these aspects to refine and tailor interventions for diverse populations.

**Contribution:**

The study contributes to the body of literature about the acceptability of patient education for HTN self-management among healthcare providers and people with HIV and HTN. Findings can be used in improving education interventions using innovative approaches.

## Introduction

While global human immunodeficiency virus (HIV) prevalence remains concerning, various projects and national governments have enjoyed some success in managing HIV and giving people living with HIV (PLWH) the opportunity to live longer. This is largely because 77% of individuals who know their status are on antiretroviral therapy (ART), 72% of whom are virally suppressed.^1,2,3,4^ However, with advancing age, exposure to protease inhibitors and direct consequences of HIVPLWH are more susceptible to developing co-morbidities; cardiovascular disease (CVD) with hypertension (HTN) being one of its most common risk factors in sub-Saharan Africa.^5,6,7^ Meta-analysis and systematic reviews in sub-Saharan Africa reported prevalence estimates of HTN in PLWH ranging from 5.2% to 50%, with its pooled prevalence being as high as 25.5% in South Africa.^8,9^ One tool that can be utilised to facilitate greater patient involvement in improving health outcomes of PLWH and HTN is patient education.^10^ It has gained momentum in promoting HIV/HTN awareness in Uganda but still needs substantial development in South Africa.^11^ Therefore, this study was undertaken to gain insight into how acceptable HTN education was to patients and healthcare providers, so that this information can assist in formulating HTN education endeavours in future.

Developing health education programmes requires an awareness of which gaps in HTN management need to be addressed. Typically, the contributing factors for the lack of self-management in South Africa are failure to diagnose, lack of patient involvement in care, inadequate treatment and lack of disease information among patients and healthcare providers.^12,13^ These factors are likely why particularly in Africa and among patients with little to no education about HTN, fewer than 40% of hypertensive individuals are diagnosed. Of those diagnosed, less than 30% receive treatment, and among those treated, fewer than 20% successfully achieve blood pressure control.^14,15^ Although South Africa’s established ART programme has reduced HIV-related mortalities in the country, these have not yet fully integrated HTN management with HIV care and PLWH often have limited knowledge about HTN.^16^

Encouraging self-management among PLWH can complement support from healthcare providers, family and the community to mitigate HTN.^12,15^ Patient education is central to self-management, enhancing knowledge about HTN for early detection and sustained control.^15^ Providing disease-specific information and lifestyle guidance improves clinical outcomes in chronic conditions like HTN.^17,18^ In South Africa, initiatives such as the Integrating HIV and hEART health in South Africa (iHEART-SA) (NCT05846503) study aim to integrate HTN care into HIV treatment at primary health care clinics in Gauteng, improving diagnosis and management in PLWH.^19^

A common feature in health education is reading material like pamphlets, posters and more recently audio-visual resources. There have been several studies, each of which focuses on different health conditions, which show that in instances where patients have accessed health educational materials, patient outcomes have shown improvement.^20,21,22^ Given that such interventions have shown promise, understanding the extent of acceptability regarding currently available HTN educational material is essential. Such insights are valuable for developing health education that meets local needs effectively.

Exploring acceptability is crucial for healthcare innovation, as it impacts programme success, trust, sustained involvement and achieving intended outcomes.^19^ Sekhon’s Theoretical Framework of Acceptability (TFA) comprising seven constructs named affective attitude, burden, perceived effectiveness, ethicality, intervention coherence, opportunity costs and self-efficacy provides insights into participants’ responses. Using TFA in interviews helps design interventions aligned with user needs.^11^ This study examines the acceptability of HTN self-management education among PLWH and HTN and healthcare providers in Johannesburg, South Africa, participating in the iHEART-SA study, described earlier.

## Research methods and design

### Study design

This was an exploratory descriptive qualitative study using in-depth interviews (IDIs) with both PLWH and HTN and healthcare providers. It was nested in a stepped-wedged cluster randomised effectiveness-implementation hybrid study (iHEART-SA [NCT05846503])

### Setting

The iHEART-SA study was conducted in Region F of the City of Johannesburg, Gauteng Province, South Africa. This urban area includes nine primary health care clinics that served as the study sites. The clinics in Region F were grouped based on patient volume (low, medium and high) to facilitate the randomised cluster stepped-wedge study design, ensuring a balanced implementation across different clinic capacities. This qualitative study was conducted in six of the nine PHC clinics participating in the iHEART-SA study. These clinics were included based on their duration of exposure to the iHEART-SA intervention. In 2019, it was estimated that each clinic served 5681 PLWH and HTN. These clinics provide a range of sexual and reproductive health services, comprehensive acute and chronic care, community outreach and HIV and tuberculosis screening and treatment services.^19^ PHCs like these are the backbone of the South African healthcare system managing majority of hypertensive PLWH.^23,24^

### Study population

Integrating HIV and hEART health in South Africa, participants who consented to be called for additional studies were invited if they were living with both HIV and HTN and on medication. Additionally, healthcare providers, including enrolled and professional nurses involved in the iHEART-SA study, were also invited to participate. For PLWH and HTN inclusion criteria were: ≥ 18 years old, able to converse in English, willing to give written informed consent, had been using hypertensive medication for 6 months or more and on ART for a year and virally suppressed. Eligible healthcare providers were those with experience in delivering health education within the selected PHCs. No participant refused to participate or withdrew from this qualitative study.

### Sampling strategy

The study was originally designed to use purposive sampling to select participants based on specific criteria. However, because of low attendance at scheduled interviews, we transitioned to convenience sampling. In this approach, only participants who were part of the iHEART-SA study met the eligibility criteria, and consented to further participation were invited to join the study as they came to the clinic to collect their chronic medication refills. Typically, 1–2 participants were interviewed per day at each clinic throughout the study period. Not all consecutive eligible participants were interviewed because of the time required for recruitment processes, conducting interviews and the rotation of data-collection visits across different clinics on non-consecutive days. Eligibility was confirmed from the iHEART-SA study database.

Healthcare providers offering iHEART-SA intervention were purposively recruited. The researcher approached these individuals after obtaining their information from the iHEART-SA research coordinators.

### Integrating HIV and hEART health in South Africa educational material

During the 6–12-month intervention phase (dependent on the step), iHEART-SA research nurses were stationed in the triage vitals room at participating clinics. Their role was to ensure the availability of functional blood pressure machines, facilitate accurate blood pressure readings, record the results and explain these results to patients. When patients completed their vital measurements, they were provided with their blood pressure readings and given information about what their reading meant. They were issued with pocket-sized colour-coded information booklets (green for normal, yellow for elevated, red for high blood pressure) providing information about the measurement and what to do to either lower or maintain their blood pressure, or to include medication adherence as well as holistic health and wellness behavioural strategies. The duration of the measurement, recording and explanation process varied depending on the patient’s level of understanding and the questions they asked. Throughout the iHEART-SA intervention phase, patients who continued to attend the clinic had their blood pressure readings taken and recorded at each visit, along with the opportunity to receive information and support from the study nurse. In the clinic there were also colour-coded posters, again with information on what different blood pressure values meant and what lifestyle change to undertake to get blood pressure under control or to maintain a healthy blood pressure. All the information material was available in English, isiZulu, isiXhosa, and Shona to cater to most of the clinic attendees.

### Data collection

Trained researchers (XS and AN) conducted IDIs using a pilot-tested semi-structured interview guide (Supplementary material A & B) that was developed using the TFA by Sekhon and included questions to respond to the constructs of affective attitude, burden, ethicality, intervention coherence, opportunity cost, perceived effectiveness and self-efficacy.^25,26^ Data were collected in private rooms at the PHCs or at the iHEART-SA research offices in cases where PHCs did not have private rooms. Interviews were conducted in English, and on average each interview lasted for 25 min. The sample size was not predetermined; instead, data collection for each group of interviewees continued until saturation was reached. Saturation was defined as the point at which no new insights or information emerged from additional participants, in accordance with the guiding theoretical framework. The IDIs were audio recorded. Data from pilot interviews were included in the final analysis.

### Data analysis

Audio recordings were transcribed verbatim, and quality checked. Initially, deductive coding was utilised to organise the data according to each TFA domain (affective attitude, burden, ethicality opportunity cost, intervention coherence perceived effectiveness, self-efficacy). Directed content analysis was then applied to identify and categorise instances of TFA domains. This process involved reading each transcript and coding occurrences related to each TFA domain using the definitions provided for each domain.^26^ Specific codes within each TFA domain were grouped into themes. Coding was done individually (XS and AN) and then discussed within the research team (STL-E and JK) to ensure intercoder reliability and consistency in the coding. MAXQDA software version 22 was used for analysis. Initial analysis was completed by the primary researcher (XS) and reviewed by other team members (STL-E and JK). The findings of this study were reported according to the consolidated criteria for reporting qualitative studies (COREQ).^27^

### Steps taken to ensure trustworthiness

The study enrolled participants from six out of nine selected clinics, which allowed for diversity across social classes, enhancing the transferability of the findings. Researchers recognised that their professional backgrounds and clinical expertise could influence the research process by creating assumptions about effective care practices. To counter this, they used a reflexive approach, documenting reflections, thoughts and potential biases in a reflexive journal during data collection. This method ensured that the study’s findings accurately reflected the experiences and perspectives of PLWH and healthcare providers. The use of reflexivity maintained the integrity and objectivity of the research. As a result, the findings are more representative and credible.

### Ethical considerations

Ethical clearance to conduct this study was obtained from the University of the Witwatersrand Human Research Ethics Committee (No. M240316). Ethics for the parent study was provided by the University of the Witwatersrand Human Research Ethics Committee (UH phase reference number: M211160) and Emory University. Permission to conduct the sub-study was provided by the iHEART-SA principal investigator. The study was conducted according to the ethical guidelines of the Declaration of Helsinki. Written informed consent was obtained prior to participation. The audio recordings and the transcripts were kept secured electronically in a password-protected folder that was accessible only to the study investigators. A unique code was allocated to each participant to ensure confidentiality; no personal identifiers were used during storage or analysis. Each person with HIV and HTN received a R150.00 reimbursement to compensate for their time, inconvenience and travel.

## Results

The findings are presented under seven constructs of the TFA: affective attitude, self-efficacy, burden, perceived effectiveness, opportunity costs, ethicality and intervention coherence. We present the results within each construct divided into distinct sub-themes. The data had minimal variation in opinion between participants. We have documented the opinions of both groups in relation to each construct, highlighting any differences that emerged. We have ensured that it is clear and concise, allowing readers to easily understand who expressed specific views and how each group felt about a particular construct/theme.

### Demographic characteristics of the participants

A total of 31 IDIs were conducted. A total of 13 patient participants with HIV and HTN participated and the majority were female (11/13); amongst these 10/13 had attended school at least up to grade 12 level. The mean age was 43.9 years with a standard deviation of 7 years. The participants had well-established conditions with an average duration of 10.5 years living with HIV and 5.8 years living with HTN. The number of participants in each of the single and married categories was 4/13. Most of the patients (8/13) had at least some type of employment (see Table 1).

**TABLE 1:** Summary of the social demographics of people living with HIV and hypertension.

Characteristics	Frequency (*n*)
**Gender**	
Female	11
Male	2
**Level of education**	
Primary	3
Matric	8
Tertiary	2
**Marital status**	
Single	4
Married	4
Widow	3
Divorced	1
Widowed	1
**Occupation**	
Employed	7
Self-employed	1
Not employed	5

*n*, number.

A total of 18 healthcare providers enrolled in the study, with the minority 2/18 being males. Healthcare providers’ ages ranged from 30 to 64 years, and the mean age was 38.7 with a standard deviation of 7.6 years. Healthcare providers had a minimum of 4 years of experience working in PHC settings in Johannesburg Region F. Professional nurses comprised the bulk of the healthcare professional sample (11/18); refer to Table 2.

**TABLE 2:** Summary of social demographics of healthcare providers in Johannesburg region F.

Characteristics	Frequency (*n*)
**Gender**	
Female	15
Male	2
Transgender	1
**Marital status**	
Single	6
Married	12
**Occupation**	
Professional nurse	11
Enrolled nurse	5
Health promoter	2

*n*, number.

### Theoretical framework of acceptability construct and themes

Eleven themes emerged from the seven TFA constructs. Three themes arose from the affective attitude and burden constructs. Emotional discomfort because of colours (affective attitude construct) and staff shortage and diagnosis denial (both under the burden construct) were identified as barriers to patient education. The remaining eight themes were viewed as facilitators of health education.

### Acceptability of patient education for hypertension self-management

#### Construct: Affective attitude

Affective attitude denotes how an individual feels (either negatively or positively) about the intervention.^26^ Two themes were identified as facilitators (simple understandable language, empowerment through knowledge) and one as a barrier (emotional discomfort because of colours) of affective attitudes towards the patient education for HTN self-management.

#### Theme: Simple understandable language

Both patients and healthcare providers welcomed the fact that irrespective of which South African language was used in the educational material, both the simplicity of the language accompanied by the graphically presented information was understandable. There was appreciation of the blood-pressure tracking table included in the booklet:

‘Everything is quite clear and simple if you give yourself time to understand what exactly the contents of the booklet are, everything is written in simple English. There are also pictures in my book which makes it easy for me to understand. Also, the booklet has a table where I write and track my blood pressure.’ (JPC-741, female, 33 years, matric)

‘So far so good. I cannot complain and what I like about it, even the information that is in the booklets, it’s very simple, straightforward, even our patients are able to understand it. Remember the information is provided in a very simplified language … Zulu, Xhosa, English.’ (HCW005, male, 34 years, Health Promoter)

#### Theme: Empowerment through knowledge

The patient education empowered PLWH to understand their condition. The booklet was viewed as being akin to a convenient reference text, which they could consult when in doubt about their health conditions. Healthcare workers shared a similar sentiment with respect to the enhancement of their HTN knowledge. Further, they found the educational material, especially in the absence of formal professional development sessions, to be a reliable source of updated information which they in turn were able to use to guide their practice:

‘It’s very [*helpful*], because if there’s something that I don’t understand, I just open the book and read. So, it serves as my reference every time … and I am very happy with the education [*I*] am getting at the clinic.’ (MVC643, female, 33 years, matric)

‘When I joined the clinic, I was not competent in offering hypertension education but through this booklet I have been able to gain information, am excited about this booklet. It has sharpened my hypertension education skills … remember guidelines change so this booklet has all information that I need to share with my patients … it has been easy to offer education now. I have learnt from this booklet. I have not attended any training but through this intervention I am empowered.’ (HCW018, female 32 years, Professional Nurse)

#### Theme: Emotional discomfort because of colours

While participants expressed their overall interest for the intervention, some noted discomfort associated with the colours of the educational materials provided. Healthcare workers revealed that patients felt that red booklets drew unnecessary attention to the recipient. Some patients found this to be so distressing, that they refused the booklet, but others put aside their fear of social exclusion in favour of the healthcare workers’ advice to focus on improving their health instead. It should be noted, however, that such discomfort was not universally perceived as a barrier to successful health education:

‘One of my clients refused to take a red booklet because he did not understand why he had to get a red one, after explaining he promised to come back better [*at the*] next visit … he refused to take the booklet because of the colour.’ (HCW001, female, 39 years, Professional Nurse)

‘The colours are a bit offside because, you know, people tend to stigmatise you when you have something that obviously is different from them, carrying the red booklet is not great, but what the nurse told me was not to focus on the colour but to understand why I got the red one and aim to reduce the BP (blood pressure).’ (MVC643, female, 33 years, matric)

### Construct: Burden

Burden refers to effort an individual thinks is required for the successful outcomes of the intervention if they participate (Table 3).^26^ There were three themes for burden: one facilitator being effort and two barriers being staff shortage and diagnosis denial.

**TABLE 3:** Acceptance of patient education for hypertension self-management. A summary of the themes and Sekhon’s theoretical framework of acceptability construct.

TFA construct	Themes	Facilitator or barrier
Affective attitude	Simple and understandable language Empowerment through knowledge Emotional discomfort because of colours	Facilitator Facilitator Barrier
Burden	Effort Staff shortage Diagnosis denial	Facilitator Barrier Barrier
Ethicality	Empowering and reduces workload	Facilitator
Intervention Coherence	Health improvements	Facilitator
Opportunity costs	Benefits	Facilitator
Perceived effectiveness	Improvement in hypertension management	Facilitator
Self-efficacy	Confidence in self-management	Facilitator

*Source:* Sekhon M, Cartwright M, Francis JJ. Acceptability of healthcare interventions: An overview of reviews and development of a theoretical framework. BMC Health Serv Res. 2017;17(1):88. https://doi.org/10.1186/s12913-017-2031-8

TFA, theoretical framework of acceptability.

#### Theme: Effort

Patient participants were initially more amenable to the guidance available from educational healthcare literature and healthcare workers than others. However, the general feeling was that the steps required to maintain acceptable blood pressure were more significant than inconveniences like having to concentrate on the information they needed to read. In fact, the desire to be able to manage their HTN was so strong that some went to great lengths to adapt their habits and monitor their own blood pressure:

‘Yes, reading the booklet requires concentration but I think it’s for my good because now I know how to manage my BP (blood pressure) … I remember one day the nurse made me stay long in his room telling me about my BP, I was annoyed because it was like he was threatening me about stroke, but after that session I felt the need to change my lifestyle … that really helped. Now my BP ranges between 130/89 and 136/92.’ (GNC494, male, 62 years, matric)

‘Oh, it’s easy. It’s easy to self-manage because you know that if it’s high, it means I’m doing something wrong. So, I must adjust what I’m doing wrong. I have bought myself a BP (blood pressure) machine just to check it at home and I record on the booklet so that my nurse can see the BP… this has helped me to know what makes it high (participant laughs) it’s relationship stress.’ (ESC748, female, 43 years, matric)

#### Theme: Staff shortage

Staff shortage resulting from over-burdened clinics was a key barrier to offering comprehensive education. Despite their eagerness to engage in the intervention, healthcare workers expressed concerns that these conditions limited the quality and consistency of health education they were able to offer patients. Without additional staff, healthcare workers would have to use their limited time dispensing medication instead of providing essential health education, reducing their ability to support patients effectively:

‘… sometimes we don’t have enough time to give health education because it’s busy. This clinic is one of the busiest in Johannesburg, sometimes it becomes hard to do other stuff such as offering the intervention. Thus, we end up focusing on only giving medication not focusing on the intervention … There is a need for more staff so that we can offer the intervention.’ (HCW006, female 44 years, Enrolled Nurse)

‘Oh, oh. I think it’s quite doable. However, as I mentioned, that the more human resource or staff, the more it’s doable … but having extra staff to focus on delivering the intervention can work.’ (HCW007, female 44 years, Professional Nurse)

#### Theme: Diagnosis denial

Despite the healthcare providers’ motivation to offer HTN education, accepting HTN diagnosis is sometimes problematic particularly for PLWH. This is either because they find it inconceivable that they could have multiple diseases concurrently, or because they view HIV as being a far more serious condition than HTN. Consequently, patients neglect their HTN management, and at times this is exacerbated by their healthcare professionals who over-focus on HIV compared to the detrimental effect of HTN. The healthcare provider reported that such an attitude towards HTN management could jeopardise the continuity of HTN patient education:

‘In some cases, it is a challenge because some of our patients do not believe that one can have both conditions. For example, a patient taking ART might not accept that they have hypertension on top of HIV. They do not concentrate on taking care of themselves when it comes to hypertension; the only thing that matters to them is HIV. So, now you are telling them their BP (blood pressure) is high, and they need to take hypertensive medication, it becomes a problem.’ (HCW002, female 35 years, Professional Nurse)

‘Patients tend to concentrate only on HIV, they don’t want to accept hypertension diagnosis. So, offering hypertension education becomes hard. I am not sure how we can work around this one going forward because what happens is that the clinicians themselves, they concentrate more on HIV than hypertension. That is why am worried about sustainability of patient education [*with regards to*] hypertension.’ (HCW002, female 35 years, Professional Nurse)

### Construct: Ethicality

Ethicality refers to the extent to which the intervention is considered a good fit with personal values.^26^ One theme was identified; patient education was found to be empowering and reduced workload.

#### Theme: Empowering and reduces workload

Educational intervention was twice as likely beneficial for PLWH and HTN as they were able to self-manage their health and build up the confidence to advocate for the programme within their communities. Healthcare providers reported to have reduced workload as their clients utilised information from the booklets and posters:

‘It helps me. It reduces the burden of me having to say one thing all the time because some patients, just by looking at the posters, they can be able to have the hypertension information they are able to take points to say … this is what I need to do to control my BP (blood pressure). It is a very good thing because it also reduces the burden on my job, you know … It fits well with my work ethic.’ (HCW017, female, 36 years, Enrolled Nurse)

Moreover, PLWH and HTN viewed the intervention so positively that they proactively shared HTN information and even suggested that the intervention should be expanded to include other clinics:

‘I have started sharing my booklet with my friends [*and*] family because I can see that this thing is working, there is a great need to have this education provided in every clinic.’ (MVC643 female, 33 years, matric)

### Construct: Intervention coherence

Intervention coherence is the extent to which participants understand the intervention and how it works.^26^ Health improvements were identified as a factor facilitating acceptance of patient education.

#### Theme: Health improvements

The health education was viewed as useful to the participants. People living with HIV and HTN acknowledged that applying the information from the sessions helped them to improve their adherence to medication, manage their blood pressure, and improve their well-being. Some expressed excitement at being able make informed health-management decisions. Patients’ claims were verified by healthcare providers who remarked that they have noted a decline in the number of PLWH with uncontrolled HTN:

‘Health education is very beneficial to me because I am now able to control my blood pressure through exercising, through cutting down some foodstuffs and eating healthy, and [*being*] able to take my medication correctly. It has been quite beneficial to me. And as I noticed last month, my blood pressure was normal. It’s really helping me a lot.’ (MVC545, female, 42 years, matric)

‘It benefited them in terms of managing their blood pressure, patients with uncontrolled BP (blood pressure) have declined. You can tell from their booklets that there is a change; people’s BP is improving compared to before the introduction of the intervention.’ (HCW002, female, 35 years, Professional Nurse)

### Construct: Opportunity cost

Opportunity cost refers to the potential loss or gain from other options when a choice is made.^26^ Participants gained more benefits from participating in the intervention. This was the only theme identified as a facilitator for acceptability of patient education for self-management.

#### Theme: Benefits

Participants found the opportunity cost of engaging in the intervention to be justified by the benefits they received. People living with HIV and HTN reported that they are open to receiving more information about HTN. Healthcare providers reported that the health education has led to reduced number of patients they see in the facilities:

‘For me, this was not a problem because I was there in the facility to do that. It works for my benefit that if people have managed BP (blood pressure), it becomes lighter even in the facilities. Do you understand that if this can be done across the country, in the long run, we might have reduced the number of people seen in the facilities? This also provided me a chance to learn new things. It allowed me to learn why people fail to take medication.’ (HCW002, female, 35 years, Professional Nurse)

Another participant shared the same sentiments:

‘There is nothing that feels [*more*] refreshing than getting my hypertension education, there is no way this can feel like wasting my time, why say that is like if you get more education [*you*] will be little more informed on different things to manage my high blood … okay I mean that I will learn and that it would make a change.’ (ESC501, female, 43 years, matric)

### Construct: Perceived effectiveness

Perceived effectiveness refers to the extent to which an intervention is expected to achieve its purpose.^26^ Participants reported that patient education facilitated an improvement in their HTN management skills.

#### Theme: Improvement in hypertension management

Provision of health education was reported to have increased awareness among PLWH and HTN. Participants reported improved health status which led to reduced clinic visits:

‘The education contributes positively to their lives, we have seen great improvement in overall health status in our patients with hypertension in this facility, the education has helped in allowing people to understand their condition.’ (HCW003, female, 31 years, Health Promoter)

People living with HIV and HTN felt the same way as healthcare providers:

‘The education is very helpful sister; my hypertension is now managed after I have been following the guidance … I find the materials to be effective. They are helpful in providing essential information and supporting my health.’ (MVC643, female, 33 years, matric)

### Construct: Self-efficacy

Self-efficacy is an individual’s confidence that they can perform the procedures of the intervention.^26^ A participant identified that they were confident in self-management.

#### Theme: Confidence in self-management

Patients view themselves as possessing high levels of confidence in their ability to manage their HTN. The healthcare professionals also displayed confidence in the patients’ HTN management competency. This newfound confidence was reflected in a patient’s ability to monitor their blood pressure, adhere to medication and make informed lifestyle choices:

‘I am very confident in managing my high blood, you know the way this change has excited me. I am now able to self-manage; can check my bp, [*and*] take my medication correctly. My booklet is my bible.’ (ESC501, female, 43 years, matric)

Healthcare providers feel confident in providing education for HTN self-management because they have positive experiences:

‘I am super confident. Yeah, I’m confident, because I have seen it working for my patients, they are doing well and staying in treatment.’ (HCW006, female, 44 years, Enrolled Nurse)

## Strategies to enhance acceptability

Apart from the TFA guided interview, all participants also responded to prompts requesting suggested strategies to improve acceptability. Healthcare professionals advocated strongly for ongoing training to be able to keep abreast of evolving guidelines and practices relating to HTN management. People living with HIV and HTN suggested including more non-traditional ways of disseminating HTN information and sustaining interest in related matters, like using multimedia and strategies. Some participants also suggested sending messages to patients. However, accessing patients’ active contact details might be an obstacle.

### Training

Healthcare providers and PLWH and HTN emphasised the importance of ongoing training to ensure they stay informed regarding HTN information. They suggested methods such as monthly workshops, waiting area screens or phone health reminders as significantly improving how well PLWH and HTN understand and use the information provided to them:

‘The workshops can be done at least once a month, or quarterly. There is a need for us to undergo training frequently to stay updated with hypertension information. Guidelines keep on changing so there is a need that we keep up with new information.’ (HCW005, male, 35 years, Health Promoter)

Participants emphasised the need for the integration of multimedia resources to reinforce educational messages, such as using screens in waiting areas to continuously display educational content:

‘… Lastly, is to have something we can watch. It will help. Because remember, when we are sitting there by the waiting area, you’re thinking about a lot of things at home, right? But if there could be something to watch related to hypertension that will allow us to have more knowledge. It will also benefit those that are not yet hypertensive.’ (GNC154, female, 39 years, tertiary)

Other participants shared that sending PLWH educational messages could be effective, although participants cited that this might be hard especially getting patients active contact details:

‘Creating a database of people with hypertension, sending them messages on hypertension will be great. You know, people like their phones. It’s easy for them to check. These messages should be structured as the booklets are. In that way, I think this intervention could work more effectively.’ (GNC494, male, 62 years, tertiary)

## Discussion

In this study undertaken in Johannesburg, South Africa, IDIs were conducted with a total of 31 participants, including PLWH and HTN along with healthcare providers to gain clarity on their acceptability of the current tools for patient education for HTN self-management. We found that there was a high acceptability among healthcare providers and PLWH and HTN alike. The identified facilitators of patient education include the use of simple and understandable language, empowerment through knowledge, reduced burden, health improvements, benefits to HTN management and increased confidence in self-management. Emotional discomfort because of colours, staff shortage and diagnosis denial were barriers of acceptability of patient education. Participants also identified sustained professional development for healthcare staff, multimedia presentations and messages communicated to patients’ mobile phone as potential areas for HTN education improvement.

The recurring responses from both patients and healthcare providers emphasised the fact that HTN education was beneficial for both groups of respondents. Patients acknowledged that the intervention empowered them to take more control over their health. The implication of such empowerment bodes well for patients in our study as the potential advantages extend beyond just encouraging regular blood pressure monitoring and medication management. Vainauskiene et al.^28^ state that patients who feel empowered to self-manage their conditions also enjoy better mental health and relationships with others. Another positive result of patient empowerment is that it reduces an over-reliance on healthcare staff in monitoring patients’ HTN. Nurses could support PLWH in a lesser amount of time and focus on other duties. Each disease in isolation of the other may not necessarily have the same positive outcomes for patients and their healthcare providers when patients must manage both conditions concurrently. Gondongwana and De Wet-Billings and Milovanovic^29^ emphasise that not all patients are able to cope with multiple treatment regimes. For instances where patients can access integrated chronic care facilities, not all health professionals have the adequate skills across a range of chronic conditions to be able to provide acceptable levels of patient support. This suggests that the results from our study when analysed in tandem with HIV self-management investigations, can be used to inform further health education solutions for PLWH and HTN.

Our study showed that simplified educational materials improved patients’ comprehension of HTN. Thereby self-management was facilitated, and patients were emboldened to share information with friends and family. People with whom these patients share information with may be encouraged to take control of their health too. This is not a far-fetched possibility, as a recent scoping review in lower- and middle-income countries suggested that stakeholders’ opinions on recommending treatment intervention to a friend or family help determine acceptability.^30^ Studies undertaken in various sub-Saharan African countries (including South Africa, Uganda, Ethiopia and Nigeria) have also shown a positive correlation between patients’ understanding of information and their willingness to share it with the greater community and subsequent uptake in disease management.^11,31,32,33,34^ Besides language, a Ugandan investigation concluded that PLWH who received structured HTN education had better adherence to medication and lifestyle modifications compared to those who did not receive HTN education.^31^ If the way information is presented as well as the accessibility of the language used is well considered, it could be beneficial to policy makers and HTN content creators particularly for South African PLWH with limited formal education, who are susceptible to high HTN-related mortality.

This study also highlighted the fact that while participants acknowledged the effort required to engage with the intervention, they perceived these demands as justified by the benefits. The balance between perceived burden and benefit is essential, as it aligns with the TFA, which posits that interventions are more likely to be accepted when the perceived benefits outweigh the costs.^26^ Our study reported inconsistencies in perceptions about the colour of the booklets used. While a few expressed emotional discomfort stemming from the belief that the colour led to stigma, others believed that the booklet’s information was more important than the colour used. This finding is supported by previous studies that emphasise the importance of perceived effectiveness in the acceptability of health interventions.^13,26,35^ The positive response to the intervention is consistent with the evidence supporting the effectiveness of patient education in managing chronic diseases.^11,22^ We are convinced that one explanation could be that our results may explain a common belief among individuals that certain situations and solutions may not be universally applicable. Although the entire iHEART-SA intervention was designed with extensive and intense stakeholder input, this study indicates that there will be no intervention which receives universal satisfaction from its recipients.

It emerged that patient education could be more acceptable if there could be continued training for health professionals, and integrating multimedia resources (sending health messages, screens displaying educational content in clinic waiting areas). These were thought to be practical strategies to reinforce health messages. Our findings are in line with what previous studies reported where implementing mobile health reminders has been acknowledged for its potential to enhance patient outcomes, and as an effective tool for engaging diverse population groups.^36,37,38^ This could serve as a better strategy as it was noted in a HTN health coaching study among PLWH in the United States, that offering a digitally based blood pressure self-monitoring programme with training, behaviour support and physician communication was acceptable, although it had to be mentioned that the utilisation decreased with time.^39^

Incorporating the messaging of health education between clinic visits could serve as a back-up because once PLWH are managed, they become eligible for multiple-month dispensing.^40^ Thus, having the option of health education in between longer visits could serve as a means of continuing patient engagement. This approach could help bridge the gap between patient visits and ensure that key information is consistently reinforced. However, barriers such as the logistical issues of maintaining accurate patient contact details for mobile reminders must be addressed. Strategies such as routine data updating during clinic visits or integrating this task into existing health information systems could enhance the feasibility of this approach.

This study provides new insights into the potential of using multimedia resources within clinic settings as a means of reinforcing educational messages. This method can be particularly effective in resource-limited settings. For example, a study that examined the impact of a digital HTN self-management and lifestyle change support programme on blood pressure in the United States reported that comprehensive digital heath on HTN and home blood pressure monitoring was effective for self-management over a year.^39^ The emphasis on regular training aligns with previous research that highlights the dynamic nature of HTN guidelines and the necessity for continuous professional development among healthcare providers.^41^ There is a need to have regular in-service trainings to ensure that healthcare providers are offering updated care.

Findings from this study highlight the need to appreciate that applying the TFA requires welcoming the overlap of themes within constructs, as such interpretation of findings requires a multi-faceted lens as they fit in more than one construct. Empowerment through knowledge, empowering and reducing workload, confidence in self-management, and improvement in HTN management. These themes highlight how participants gained knowledge, confidence and empowerment through patient education. Although these themes are framed under different constructs, they are focused on the benefits of knowledge leading to better management and confidence. This finding is consistent with what other studies reported on the issues of overlap while using this framework.^11,13,42^ This overlap could be because this research explored concurrent acceptability, thus assessing all seven constructs.

## Strengths and limitations

This is the first study of its kind in South Africa that informs about the acceptability of patient education offered by iHEART-SA. Several important strengths were noted. Firstly, our study explored concurrent acceptability and enabled participants to suggest strategies to enhance the acceptability of the intervention, for modifications in intervention delivery. Secondly, we recruited participants from different PHC facilities to judge acceptability from various locations. Thirdly, we interviewed stakeholders in the provision of HTN care thus getting perspectives from both patients and providers. Our study had some limitations. It included more female participants than male participants among both PLWH and HTN patients, as well as healthcare providers, consistent with the typical gender composition in the area. It is uncertain whether sex differences may affect acceptability of patient education. Further, a large majority of the patient population had completed formal secondary schooling with a few having received higher education. This means that it is possible that patients with no to very little education or low literacy levels may not be able to engage with the educational material with the same level of understanding as our study participants. At recruitment, we unintentionally interviewed a group of patient participants with established HIV and HTN. Being experienced with chronic conditions may have influenced their responses compared to those newly diagnosed. Additionally, the convenience sampling strategy limited inclusion of certain groups (e.g., stable chronic patients with multi-month scripts, men, youth and very old people) particularly among patient participants, potentially affecting the diversity of perspectives.

## Conclusion

The educational booklets and posters were determined to be acceptable by both healthcare providers and PLWH and HTN. The results suggest that successful implementation of patient education is feasible and appreciated. Additionally, innovative integration of continued education and training for HTN self-management is critical in HIV programming. Programme designers need to improve on this by tackling the recognised barriers to implementation and leveraging technology. Finally, there may be a need to restructure the TFA as there is a noticeable overlap of themes within constructs. Future research should further explore these aspects to refine and tailor interventions for diverse populations, contributing to more effective health-education strategies that can be broadly applied.
